# Applying an educational program for primigravida women regarding their health and wellbeing

**DOI:** 10.1186/s12884-026-09423-4

**Published:** 2026-07-17

**Authors:** Noura Adel Sayed Hassan, Nawal Mahmoud Soliman, Mona Abo Bakr Abdellatif, Fatma Gomaa Mohamed

**Affiliations:** Family and Community Health Nursing, Faculty of Nursing, Ain Shams University, Cairo, Egypt

**Keywords:** Primigravida, Maternal Well-being, Pregnancy, Educational Program, Health Promotion

## Abstract

**Background:**

Primigravida women's health and well-being during pregnancy are crucial as they promote healthy fetal development and birth outcomes, reduce complications for both mother and baby, and positively impact the whole family. Primigravida is a term referred to a woman who is pregnant for the first time. This study evaluated an educational program’s effect on health knowledge and well-being among primigravida women.

**Methods:**

A quasi-experimental design was conducted with 95 primigravida women in Cairo. The intervention involved six weekly sessions (45–60 min) based on the Health Promotion Model. Pre- and post-tests assessed knowledge and well-being. Cohen’s d was used for effect size.

**Results:**

Post-intervention, participants' high knowledge levels significantly increased from 26.3% to 74.7% (*p* < 0.001). Similarly, high well-being levels improved from 10.5% to 71.6% (*p* < 0.001). A strong positive correlation (r = 0.65, *p* < 0.001) was established between health-related knowledge gains and overall well-being scores following the program implementation.

**Conclusion:**

The structured educational program effectively improved maternal knowledge and well-being among primigravida women. Integrating such tailored interventions into primary healthcare services is essential for enhancing maternal outcomes and promoting a positive pregnancy experience.

**Supplementary Information:**

The online version contains supplementary material available at 10.1186/s12884-026-09423-4.

## Introduction

Pregnancy is a transformative life stage characterized by profound physiological and psychological shifts that necessitate specialized care [14]. For primigravida women, these changes often present significant challenges due to their inherent inexperience and lack of prior health-related knowledge. This 'knowledge gap' frequently manifests as anxiety and difficulty in managing minor pregnancy discomforts, which can negatively impact both maternal and fetal outcomes [4].

While the World Health Organization (WHO) and the United Nations’ Sustainable Development Goals (SDG 3) emphasize the promotion of holistic well-being and the reduction of maternal mortality [17, 21], achieving these targets in low-resource settings remains a challenge. Despite the global mandate, there is a noticeable scarcity of structured, theory-based educational interventions specifically tailored for primigravida women in local primary healthcare contexts.

Current evidence suggests that well-being is a multifaceted construct encompassing emotional stability, social functioning, and life satisfaction [10]. However, traditional prenatal care often focuses on clinical monitoring, potentially overlooking the educational and psychological empowerment of the mother. Community health nurses are uniquely positioned to bridge this gap through systematic educational programs [22]. Yet, more evidence is needed to evaluate how these programs directly translate to improved well-being among first-time mothers. Therefore, this study aims to evaluate a structured educational program designed to empower primigravida women and enhance their health-related well-being."

### Significance of the study

The significance of this study is underscored by the unique demographic and clinical challenges within the Egyptian healthcare context. Primigravida women constitute approximately 24–28% of all pregnancies in Egypt [20], representing a high-risk group due to their lack of experiential knowledge. This vulnerability is manifested in high rates of preventable complications; for instance, local evidence indicates that gestational anemia affects nearly 44.5% of pregnant women in Egyptian primary care settings [7], while antepartum depression and anxiety are prevalent among primigravida women due to inadequate psychosocial support [13]. By targeting these specific issues—nutritional deficits, psychological distress, and the mismanagement of minor discomforts—this study directly aligns with the United Nations’ Sustainable Development Goal (SDG 3) to reduce maternal morbidity. Despite existing literature, a critical knowledge gap persists regarding the integrated impact of education on the psychosocial well-being of Egyptian primigravida women. While previous research has predominantly focused on knowledge acquisition as an isolated variable, this study addresses this conceptual fragmentation by examining the synergistic relationship between health literacy and subjective well-being. Consequently, this research provides a localized, evidence-based framework essential for empowering women and optimizing maternal nursing interventions in resource-limited primary care settings.

## Aim of the study

This study aims to evaluate the effectiveness of aneducational program on the health-related knowledge and well-being of primigravida women. This was achieved through the following objectives:

1. Assessing primigravida women’s knowledge regarding their health during pregnancy pre- and post-intervention.
2. Assessing primigravida women’s levels of well-being pre- and post-intervention.
3. Determining the correlation between the women's knowledge scores and their well-being levels after participating in the program.

### Research hypothesis

- H1: There will be a significant improvement in the mean knowledge scores of primigravida women regarding their health during pregnancy after attending the educational program compared to their pre-intervention scores.
- H2: There will be a significant improvement in the mean well-being scores of primigravida women post-intervention.
- H3: A significant positive correlation will exist between primigravida women’s health-related knowledge and their levels of well-being following the implementation of the educational program.

Previous interventions for primigravida women have largely focused on enhancing specific clinical knowledge, such as breastfeeding techniques or nutritional adherence [9, 12]. However, these programs often exhibit a methodological narrowness by failing to address the psychological burden and the overall well-being of the mother as an integrated outcome. While some recent studies have begun to explore the emotional aspects of pregnancy [5], there is a notable lack of evidence-based frameworks that combine comprehensive health education with psychological support in the Egyptian healthcare system. This study addresses these limitations by providing a holistic educational model that treats maternal knowledge and subjective well-being as interdependent variables, thereby offering a more robust and culturally specific approach to antenatal nursing care."

## Methods

A quasi-experimental (one-group pre-test/post-test) design was adopted. This design was chosen due to the feasibility of implementing the educational program within the specific clinical setting and to ensure that all eligible primigravida women during the study period received the intervention for ethical reasons. The study was conducted over six months (January to June 2025), following a sequence of three phases: assessment, implementation, and evaluation,ebrua

### Setting

The study was conducted at the Al-Marj Al-Sharqiya Primary Healthcare Center in East Cairo. This facility was purposively selected through a multi-stage process:

First, the Cairo governorate was identified.Second, the Al-Marj district was selected as a high-density urban–rural setting.Finally, this specific center was chosen because it is the largest healthcare facility in the region, serving a diverse and high-volume population of pregnant women.The center provides essential maternal services, including routine antenatal care (ANC), immunization, and health counseling. Its high patient flow rate compared to neighboring facilities ensured a representative and feasible environment for evaluating the educational program.

### Type and size of sample

Multi-stage sampling technique was employed to select the study setting and participants. In the first stage, the Cairo Governorate was partitioned into four main geographical zones (North, South, East, and West), totaling 73 family health centers. In the second stage, the East Cairo zone (containing 27 centers) was purposefully selected. From this zone, the largest healthcare center was specifically chosen as the study setting because it serves a high volume of diverse primigravida women, ensuring a broad representation of the target population.

Following the selection of the setting, a sampling frame of (*N* = 125) primigravida women was identified through antenatal care registration logs. To determine the necessary sample size for statistical power, the Stephen-Thompson formula was applied, yielding a required sample of 95 participants (95% confidence level, 5% margin of error). Finally, a purposive sampling approach was used to recruit women from the identified frame who strictly met the inclusion criteria (e.g., primigravida status and gestational age), ensuring they were the most suitable candidates for the educational intervention." The sample size was calculated using the Stephen–Thompson formula to ensure a 95% confidence level and a 5% error.

$$\frac{N\times p(1-p)}{\lbrack N-1\times\left({d}^2\div{Z}^2\right)\rbrack+P(1-P)}$$

$$\mathbf{n}=\frac{125\times0.50(0.5)}{\left[125-1\times\left(\left(0.05\right)^2\div\left(1.96\right)^2\right)\right]+0.50(0.50)}=95\;\mathrm{primigravida}\;\mathrm{women}.$$

where (N) is the sample size of total primigravida women, (Z), is the standard score for the significance level (0.05),the level of confidence (0.95) is equal to (1.96),and (d) the error rate is equal to (0.05) and the probability value is (0.50) [19].

### Data collection tools

This study was carried out using two tools. After analyzing recent relevant literature and consulting experts in the family and community health nursing department.

first tool:A structured interviewing questionnaire, it's a comprehensive tool designed to collect sociodemographic data, medical history, and assess the participants' knowledge. It's composed of three parts as follows:

- Part I:
  - ➢ Primigravida women's sociodemographic characteristics include age, family income, place of residence, employment, educational level, and crowding index.

- Part II:

Previous medical, surgical, and obstetric history of primigravida women including last menstrual period, expected delivery date, BMI, minor discomfort, current pregnancy problems, and use of medications or supplements during pregnancy. To ensure data validity and minimize recall bias, the medical and obstetric history collected during the structured interviews was cross-checked and supplemented by reviewing the participants' official medical records and antenatal registration logs maintained at the health center. This ensured the accuracy of clinical parameters such as gestational age.

- Part III

Knowledge of primigravida women that was a researcher-developed tool synthesized from several validated sources [9, 15]. The final version was culturally adapted for the Egyptian context, incorporating local terminology and health practices, and was validated by a panel of experts to ensure its suitability for primigravida women in Egypt."

#### Scoring system

The total knowledge questions are composed of 30 questions. Each correct answer was given a score of one, and incorrect answer and "don't know" were given a sore of zero. A total score if total knowledge was 30. The source of information about pregnancy was excluded from the scoring. The scores were summed and converted into percentages.

### Statistically it was classified into 3 categories

- High level of knowledge if the score was ≥ 75%.
- Average level of knowledge if the score was 50≥75%.
- Low level of knowledge if the score was<50%.

Tool II: Primigravida women's wellbeing scale adapted from [3].

(A preliminary assessment of the Well-being in Pregnancy (WiP) questionnaire) and adjusted by the investigator to suit the investigation.

#### Scoring system

Possible responses from primigravida women were measured via a 3-point Likert scale (always = 3, usually = 2, rarely = 1).

### These responses were statistically classified into 3 categories

- High wellbeing if the response rate was ≥ 70%.
- Moderate well-being if the response rate was 50 > 70%.
- Low well-being if the response rate was less than 50%.

*** The scale was reversed for negative statements.

"A total score for the WiP questionnaire was calculated by aggregating responses across both dimensions. For the 'Negative Feelings' dimension (Items 17–19), reverse scoring was applied where 'Always' scored 1 and 'Never' scored 5. Consequently, higher total aggregate scores indicated higher levels of maternal well-being."

### Operational design

This phase includes a preparatory phase, content validity, pilot study and field work.

- Preparatory phase:

In order to get in-depth information about the study, this review comprised a review of recent, current, national, and worldwide relevant literature as well as theoretical understanding of many areas of the study utilizing books, papers, scientific journals, and the internet.

b) Content validity:

Content validity was performed for the tools by a panel of 3 expert professors in the Family and community Health Nursing department at the Faculty of Nursing, Ain Shams University to test the tools for appropriateness, clarity relevance, applicability and comprehensiveness. Minor modifications were performed based on their feedback.

c) Content reliability:

The previous tools were tested via Cronbach’s alpha reliability analysis

**Table:** 

Items	Alpha Cronbach	F	*P*-value
Total knowledge score	0.845	24.613	< 0.001*
Pregnant women' s well-being	0.813	19.471	< 0.001*

### Tools reliability and pilot study

"As shown in the table above, the Cronbach’s alpha coefficients for the knowledge scale and well-being scale were 0.845 and 0.813, respectively. According to Nunnally and Bernstein (1994), a Cronbach’s alpha of 0.70 or higher indicates good internal consistency and reliability. Therefore, the tools used in this study demonstrate high reliability and are suitable for academic research. Regarding the pilot study, ten primigravida women (10% of the total sample) participated to evaluate the tools' applicability and clarity. The average time required to complete the questionnaire was 30–40 min. Since no major modifications were required, the pilot participants were excluded from the main study sample (*n* = 95) to ensure that the final results were not influenced by prior exposure to the program materials. The pilot study confirmed the feasibility of the session timing and the clarity of the Arabic booklet.

### Ethical consideration

Ethical approval was obtained from the Scientific Research Ethics Committee, Faculty of Nursing, Ain Shams University (Approval Code: 24.12.438). A written informed consent was obtained from each participant after a full explanation of the study's aim, duration (6 weeks), and session frequency. The consent form also included a guarantee of data confidentiality and anonymity, ensuring that sensitive medical and demographic information would be protected and used exclusively for scientific research. Participants were informed of their right to withdraw from the study at any time.

### Program construction

The intervention was systematically conducted through five integrated phases to ensure methodological and pedagogical rigor:

The program content and study tools were developed after reviewing recent national and international literature over three weeks.

1. Preparatory & Validation Phase: The program content was developed based on a comprehensive review of recent national and international literature. To ensure Content Validity, the program’s curriculum and educational materials were reviewed by a panel of three independent experts in Family and Community health Nursing. A Pilot Study was conducted on 10% of the sample (*n* = 10, excluded from the main study) to verify the clarity of the Arabic booklet and the feasibility of the session timing.
2. Assessment Phase: A baseline pre-test was administered to evaluate the primigravida women’s knowledge and well-being using the structured study tools. This phase identified the specific educational needs of the participants.
3. Planning & Implementation Phase: Grounded in the Health Promotion Model, the program consisted of six sessions (4 theoretical and 2 practical), delivered over six consecutive weeks (one 45–60 min session per week). To foster interaction, participants (*n* = 95) were divided into five small groups (approx. 19 women/group). This group size was strategically chosen to facilitate effective group dynamics, encourage the exchange of experiences among primigravida women, and ensure sufficient time for the researcher to provide individualized feedback during the practical sessions."The sessions covered essential maternal topics: personal hygiene, nutrition, physical activity, sleep, vaccinations, and sexual health. Teaching strategies included brainstorming, role-playing, and educational videos, supported by illustrated Arabic booklets.
4. Fidelity & Monitoring Phase: To maintain consistency, the researcher utilized a standardized manual and unified educational media (laptops, posters, handouts) across all groups. Each session began with a review of previous content and concluded with a summary of key points to ensure cognitive reinforcement.
5. Evaluation & Follow-up Phase: The immediate impact was evaluated through post-testing. Additionally, a follow-up assessment was conducted one month post-intervention to evaluate knowledge retention and the sustained application of well-being strategies in daily life.

### Administrative design

To help with data collecting and program execution, an official consent letter was acquired from the dean of Ain Shams University's nursing college and presented to the general manager of the aforementioned setting.

#### Field work

It was conducted over six months, from January to June 2025, et al.-Marj Al-Sharqiya Primary Health Care Center in East Cairo. After obtaining official approval and informed consent, the researcher met with the center’s manager to explain the study objectives and schedule. Data collection and program implementation took place twice weekly (Mondays and Wednesdays) from 9:00 a.m. to 1:00 p.m. The fieldwork included three consecutive phases: Pre-intervention phase, baseline data collection from 95 primigravida women, implementation phase for delivery of the educational sessions and evaluation phase that was post-intervention assessment to determine program effectiveness. All sessions were conducted in the center’s waiting area, and confidentiality of participants’ information was strictly maintained.

### Statistical design

Data were analyzed using IBM SPSS Statistics version 26.0 (Armonk, NY: IBM Corp.). The normality of data distribution was verified using the Shapiro–Wilk test. Quantitative data were expressed as Mean and Standard Deviation (\text{Mean} \pm \text{SD}), while qualitative data were presented as frequencies and percentages.

To compare the same group across different time periods (pre and post-intervention), the Paired t-test was employed for normally distributed variables, and the Wilcoxon Signed-Rank test was used for non-parametric data. The Chi-square (X^2) test was used for comparing qualitative variables. Correlations between quantitative variables were assessed using Pearson’s correlation coefficient (r). The magnitude of the program's effect was assessed through the significance of improvements in knowledge and well-being scores (*p* < 0.05) and the strength of the correlation coefficients (r)."

## Results

Table 1 Reveals that 69.5% of primigravida women ages ranged from20 ≤ 30 years with a mean age of 26.44 ± 3.52.Also 41.1% of them had secondary education and 73.7% of them were housewives. With respect to the place of residence, 88.4% of them lived in rural areas followed by 71.6% who had sufficient income and for the crowding index 86.3% who were not crowded.

**Table 1:** Frequency and percentage distribution of the studied primigravida women according to their socio-demographic characteristics (*n* = 95)

Sociodemographic characteristics	n	%
Age		
20 < 30 years	66	69.5
30 < 40 years	25	26.3
40 ≤ 45 years	4	4.2
Mean ± SD	26.44 ± 3.52	
Education		
Primary education	10	10.5
Preparatory education	16	16.8
Secondary education	39	41.1
University or higher education	30	31.6
Employment		
Housewife	70	73.7
Working	25	26.3
Residence		
Urban	11	11.6
Rural	84	88.4
Income		
Sufficient	68	71.6
Not Sufficient	27	28.4
Crowding index		
≤ 2 Not crowded	82	86.3
> 2 crowded	13	13.7

*n* = frequency; % = percentage

Table 2 Clarifies that 62.1% of primigravida women suffer from anemia. As for their (WHO BMI categories), 54.7% of them were overweight according to the weights that they reported to me. With respect to minor discomfort and health problems associated with the current pregnancy 25.3% had heartburn. In addition 30.5% of them take nutritional supplements such as folic acid.

**Table 2:** Frequency and percentage distributions of the studied primigravida women according to their present medical history (*n* = 95)

Medical history	n	%
A diseases suffer from		
Blood pressure	15	15.8
Diabetes	4	4.2
Anemia	59	62.1
None	17	17.9
Previous surgeries		
Tonsillectomy	2	2.1
Appendectomy	10	10.5
None	83	87.4
BMI (WHO) [23]		
18.5–24.9 (normal weight)	15	15.8
25–29.9 (over weight)	52	54.7
30 and above (obesity)	28	29.5
Minor discomfort and health problems associated with the current pregnancy		
Dizziness	14	14.7
Headache	6	6.3
Heartburn	24	25.3
Frequent urination	10	10.5
Continuous vomiting	12	12.6
Varicose veins	4	4.2
Constipation	21	22.1
Bleeding	4	4.2
Medications taken		
Nutritional supplements such as folic acid	29	30.5
Nutritional supplements such as iron	20	21.1
Nutritional supplements such as calcium and vitamin D	27	28.4
Antihypertensive medications	15	15.8
Diabetes or hyperglycemic medications	4	4.2

*n* = frequency; % = percentage

Percentages may not sum to 100% because categories are not mutually exclusive; participants could report more than one condition

**Fig. 1:**
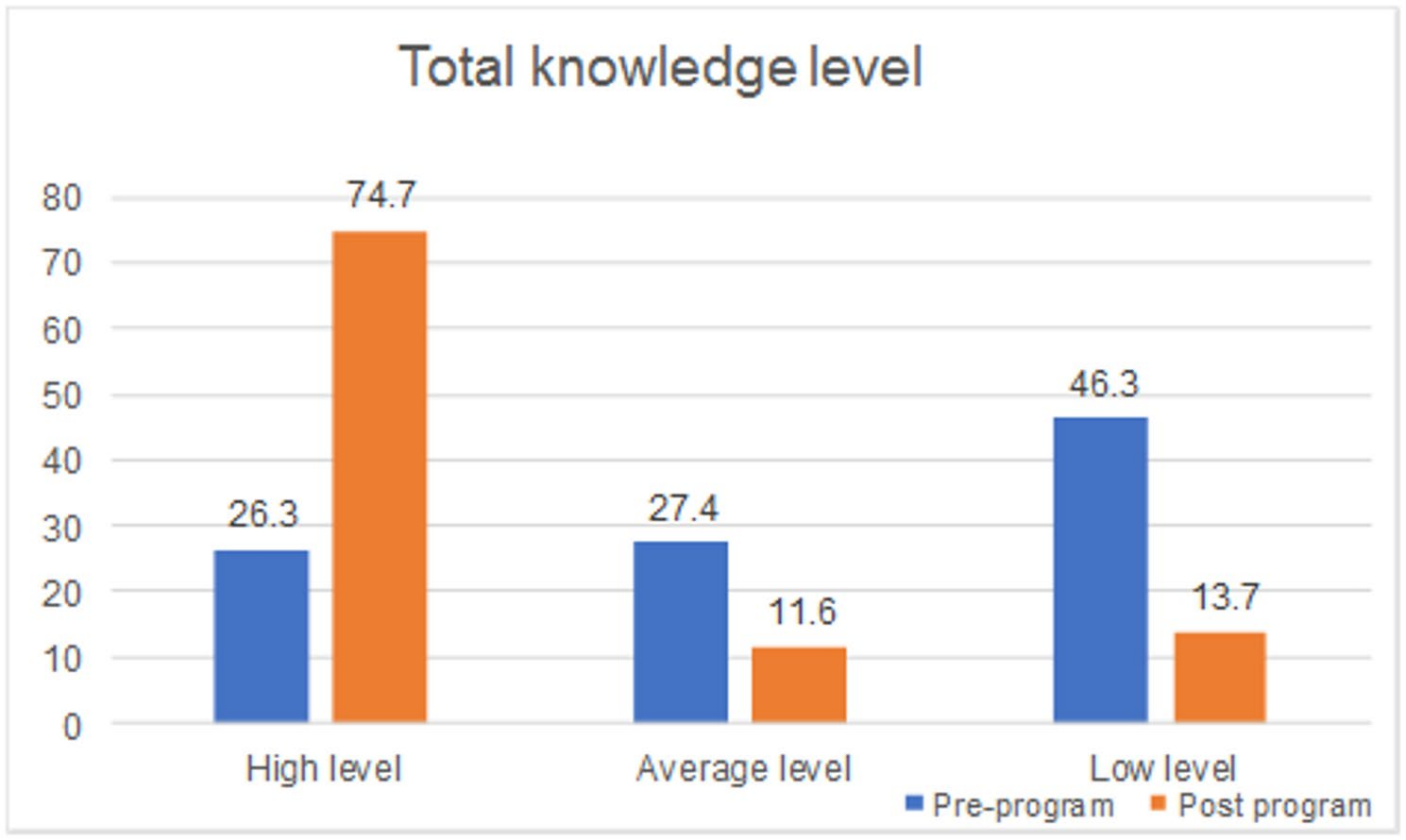
It illustrates a marked improvement, with the percentage of women having a high knowledge level increasing from 26.3% pre-intervention to 74.7% post-intervention (*p* < 0.001, t = 15.68)

Figure 1 Distribution of the studied women according to their total knowledge levels pre and post-program implementation (*n* = 95)

Knowledge levels of the participants (Y-axis represents percentage %) Percentage %).

**Fig. 2:**
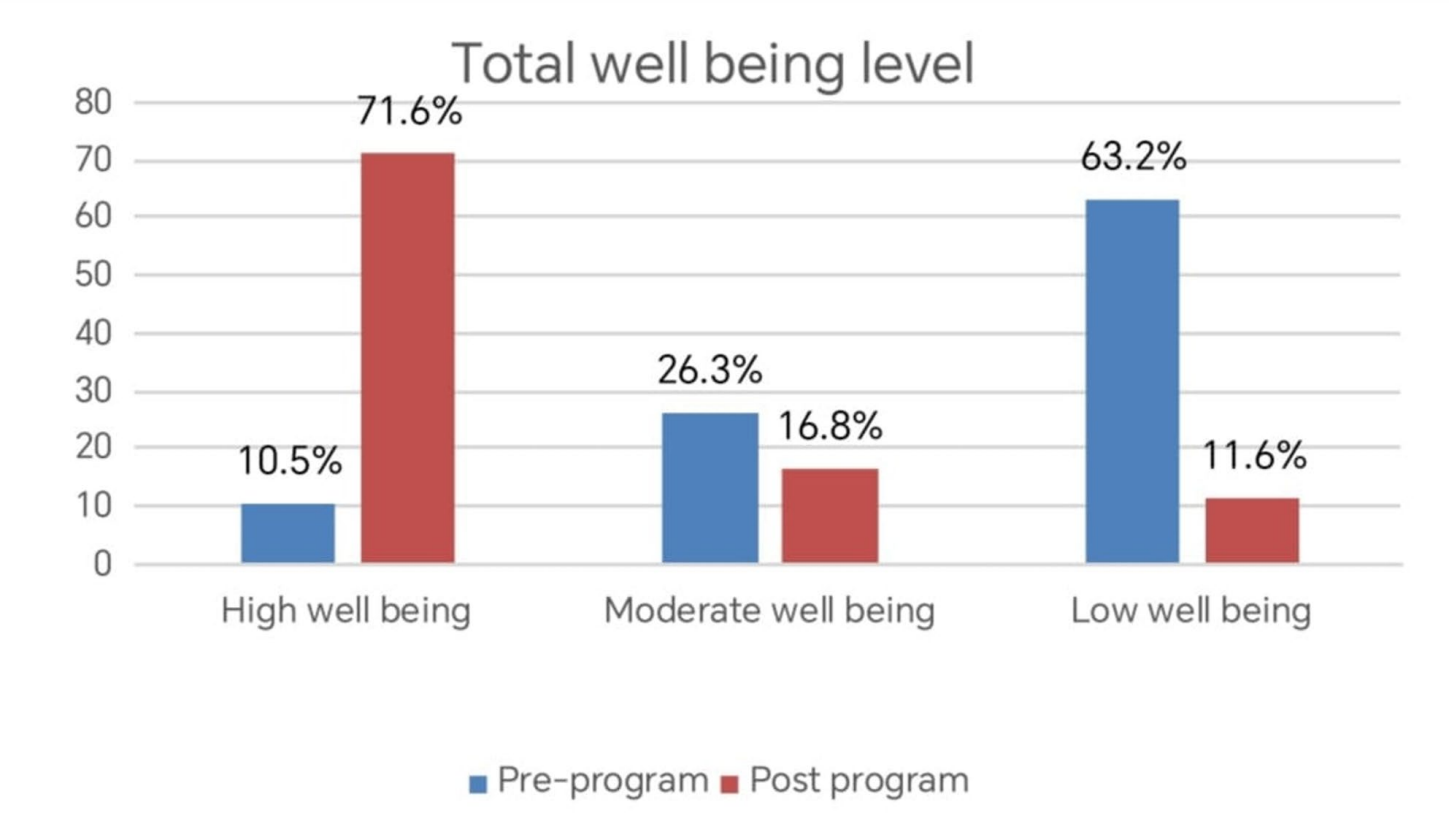
Comparison of total well-being levels among the studied women before and after the educational program (*n* = 95)

Figure 2 It shows a significant shift toward higher well-being, as high levels rose from 10.5% to 71.6%, while low well-being levels decreased from 63.2% to 11.6% (*p* < 0.001, t=12.42).

Table 3 Demonstrates that there was a statistically significant positive correlation between total knowledge scores and total well-being scores both pre-intervention (r = 0.855, *P* < 0.001) and post-intervention (r = 0.815, *P* < 0.001).

**Table 3:** Correlation between total knowledge and total well-being scores pre and post-program implementation (*n* = 95)

Total well-being	Total knowledge	
	*r*	*P*- value
Pre program	0.855	< 0.001*
Post program	0.815	< 0.001*

^*^statistically significant difference at *P* < 0.05

## Discussion

The current study evaluates the impact of an educational program on the knowledge and well-being of primigravida women, aligning with the third Sustainable Development Goal (SDG) for improving maternal health.

### Socio-demographic characteristics (Table 1)

#### Sociodemographic characteristics

The findings revealed that the mean age of participants was 26.44 \pm 3.52 years, with over two-thirds aged between 20–30 years. This aligns with [2] and reflects the peak biological and sociocultural age for first-time pregnancy in the Arab region. Regarding residence, the majority were from rural areas, consistent with ElAshmawy et al. [6].

In contrast, [16] reported a predominance of urban and highly educated participants in Romania. This discrepancy is likely rooted in the distinct sociocultural fabric of the Al-Marj region in Egypt, where rural-to-urban migration patterns and traditional educational barriers for females still influence demographic profiles. Furthermore, while residence often dictates access to resources, our findings suggest that in the Egyptian context, primigravida benefit from a robust 'collective care' model and extended family support networks that transcend geographical boundaries (urban vs. rural). This cultural safety net provides a uniform level of emotional support, effectively mitigating the impact of residence on maternal well-being compared to the more individualistic settings observed in Westernized societies.

### Economic status & medical history

Over two-thirds of the women reported sufficient income, aligning with [13] but differing from ElAshmawy et al. [6]. This variation suggests that financial security in our sample is closely tied to the husband’s stable occupational status, providing a less congested living environment. Anemia affected over half of the participants, a finding supported by [7]. Methodologically, this high prevalence in our Egyptian cohort can be attributed to nutritional habits and inconsistent adherence to iron supplementation, which remains a public health challenge in primary care settings. Regarding BMI, over half were overweight, consistent with [1]. This trend suggests a need for increased awareness of healthy lifestyles to replace traditional reliance on anecdotal advice from inexperienced social circles.

### Educational program impact

The program significantly improved knowledge levels from 25 to 75%, mirroring results by [11] and [8]. Furthermore, high well-being levels increased from less than one-quarter to over two-thirds post-intervention. This is consistent with [18].

The strong positive correlation observed between knowledge and well-being suggests a synergistic relationship. We hypothesize that increased knowledge served as a primary driver for enhanced well-being; by empowering primigravida women with practical health information, the intervention likely reduced pregnancy-related anxiety and fostered a sense of control. However, the relationship may be bidirectional, as improved psychological well-being can enhance cognitive engagement and motivation to learn. Additionally, the social support provided during the group sessions may have functioned as a confounding catalyst, simultaneously improving both knowledge acquisition and emotional stability."

The success of the program lies in its "tailored approach" to the specific anxieties of primigravida women. By addressing the psychological fear of the unknown through structured education and stress-management techniques (e.g., mindfulness), the intervention effectively transitioned participants from a state of overwhelm to one of empowered confidence.

## Study limitations

This study provides valuable insights into maternal health education in a primary care setting. However, several limitations must be acknowledged. First, the use of a quasi-experimental (one-group pre-test/post-test) design lacks a control group, which may limit the ability to control for all extraneous variables. Nonetheless, the specificity of the knowledge gains following the intervention suggests the program was the key driver of change. Second, while medical history was cross-verified with clinical records, the study partially relied on self-reported data for subjective well-being aspects, a common limitation in psychosocial nursing research. Third, as the study was conducted at a single health center, the generalizability of the findings to different geographical or socio-economic contexts may be limited. Future research should consider multi-center designs with a control group and a longer follow-up period to evaluate sustained behavioral changes throughout the postpartum period**.**

## Conclusion

### On the light of the findings of the present study, it can be concluded that

The educational program is highly effective in enhancing both health-related knowledge and overall well-being among primigravida women. Following the intervention, a substantial majority of participants achieved high knowledge and well-being levels, with a significant positive correlation established between these two variables. These findings suggest that tailored educational interventions have the potential to enhance maternal health awareness and psychological stability during the first pregnancy. While these findings are encouraging, they should be interpreted within the context of the study’s design. Practically, providing structured education in primary care settings could serve as a valuable supplement to routine antenatal care. Further research with randomized controlled trials is recommended to confirm these causal links and to inform broader maternal health policies."

## Recommendations and Implications for practice

### Based on the study findings, the following recommendations are proposed to enhance maternal healthcare services

- Clinical Practice: Structured health education programs, grounded in theoretical frameworks like the HPM, should be integrated into routine prenatal visits at primary healthcare centers. This ensures that primigravida women receive standardized, evidence-based guidance.
- Educational Materials: Development of context-specific, illustrated manuals and digital resources is essential to support health literacy. These materials should be designed to bridge the gap for women with varying educational backgrounds.
- Community & Digital Outreach: Health authorities should utilize digital platforms and community-based workshops to foster early awareness of healthy pregnancy behaviors, reaching women even before their first antenatal visit.
- Multidisciplinary Policy: Policymakers should encourage a multidisciplinary approach that integrates psychological well-being and nutritional counseling into standard maternal care protocols, rather than focusing solely on clinical parameters.
- Future Research: To address the limitations of the current study, further longitudinal research with larger, randomized samples is recommended to evaluate the long-term impact on both maternal and neonatal outcomes.

## Supplementary Information

Supplementary Material 1.

## Data Availability

The materials and data used in this study can't be accessed to the public due to reasonable request they can be obtained from the corresponding author.

## Abbreviations

EKB: Egyptian Knowledge Bank
SDG: The Sustainable Development Goals
UN: United Nations
BMI: Body mass index
n: Number
REC: Research Ethics Committee
WHO: World Health Organization
