# Supplementary material for: Applying an educational program for primigravida women regarding their health and wellbeing
Source: BMC Pregnancy Childbirth. 2026 Jul 17;26:788. doi: 10.1186/s12884-026-09423-4 (PMC13378162; doi:10.1186/s12884-026-09423-4)
Supplement: Supplementary file 1 — Supplementary Material 1. [file 12884_2026_9423_MOESM1_ESM.docx]

**Questionnaire form**

**To evaluate the effect of an educational program for primigravida women regarding their health and well-being**

**First: Sociodemographic data of primigravida women**

**Age**: A- 20<30 years ( ) B- 30<40 years ( ) C- 40≤45 years( ) **1-**

**2-Educational level:**

A- Illiterate ( ) B- Reads and writes ( )

C- Primary education ( ) D- Preparatory education ( )

E- Secondary education ( ) F- University or higher education ( )

:**3- Employment**

A - Housewife ( ) B – working ( )

**4-Place of residence:**

A - Urban ( ) B – Rural ( )

**5-Family monthly income:**

A - Sufficient ( ) B - Not sufficient ( )

**6- Number of family members = , Number of rooms =**

**Crowding index =-**

A- ≤ 2 Not crowded ( ) B - > 2 crowded ( )

**Second: Present medical ,previous surgical history, and current pregnancy history of the primigravida women**

**7-Which of the following diseases do you suffer from?**

A- Blood pressure ( ) B- Diabetes ( ) C- Liver ( ) D- Heart ( )

E- Kidney ( ) F- Anemia ( ) G- Other diseases mentioned ( )

H- None ( )

**8-Which of the following surgeries had you had previously?**

A- Kidney stone surgery ( ) B- Tonsillectomy ( )

C- Appendectomy ( ) D- Other surgery mentioned ( ) E- None ( )

**? 9-What is the date of your last menstrual period**

**? 10-What is the expected day of delivery**

**11-A) what is your current weight? , How tall are you**

**? 12-What is your body mass index**

A) 18.5-24.9 (normal weight) ( ) B) 25-29.9 (over weight) ( )

c) 30 and above (obesity) ( )

**13-What are minor discomforts and health problems associated with the current pregnancy?**

A- Dizziness ( ) B- Headache ( )

C- Heartburn ( ) D- Frequent urination ( )

E- Continuous vomiting ( ) F- Varicose veins ( )

G- Constipation ( ) H- Bleeding ( )

**14-Which of the following medications do you take during pregnancy?**

A- Nutritional supplements such as folic acid ( )

B- Nutritional supplements such as iron ( )

C- Nutritional supplements such as calcium and vitamin D ( )

D- Antihypertensive medications ( )

E- Diabetes or hyperglycemic medications ( ) - Others mentioned( )

**Third: Evaluating primigravida women's knowledge about health during pregnancy**

**Note: You can choose more than one answer**

**Primigravida women' knowledge about health:**

**? 1-What is the concept of health during pregnancy**

A - It is a state of good balance between the physical, emotional and psychological health of the pregnant woman ( )

B - It is a state of balance between the symptoms of pregnancy and the methods of treating these symptoms ( )

C - It is feeling physically good ( )

D - It is the ability to make an effort during pregnancy ( )

E - I don't know ( )

**? 2-What is the definition of healthy behavior during pregnancy**

A - Activities those pregnant women do to recover from diseases ( )

B - Activities and steps that healthy people do to prevent the occurrence of diseases ( )

C - It is the healthy lifestyle that pregnant women maintain ( )

D - I don't know ( )

knowledge of primigravida women about personal hygiene

**? 3-What is the importance of personal hygiene for primigravida women**

A - Maintaining the health of the mother and fetus ( )

B - Avoiding infection ( )

C - both A& B ( ) D - I don't know ( )

**? 4-How often should teeth be brushed**

A - Only once a day ( ) B - After each meal ( ) C - At least twice a day ( )

D - Before bed only ( ) E - I don't know ( )

**5-What are the best daily behaviors that should be followed to maintain the cleanliness of pregnant women?**

A- Daily shower ( ) B- Use of strong chemical products ( )

C- Use of appropriate vaginal products ( ) D- I don't know ( )

**? 6-How to clean the genital area**

A- Gently wash it with warm water and mild soap from front to back ( )

B- Scented vaginal products are used ( )

C- Vaginal antiseptics are used ( )

D- I don't know ( )

**7-What are the healthy habits that a pregnant woman should follow to avoid infection during pregnancy?**

A- Avoid kissing and sharing bathroom tools with others ( )

B- Wash hands with soap before eating and after using the bathroom ( )

C- Avoid wearing cotton underwear ( )

D- I don't know ( )

Knowledge of primigravida women about nutrition during pregnancy

**8-What are the specifications of a balanced meal?**

A- It is a filling and delicious meal ( )

B- It is a meal that contains all types of food ( )

C- It is a meal that contains all nutritional elements such as proteins ,

Carbohydrates, fats, vegetables and fruits ( )

D- I don't know ( )

**? 9-How many meals should a primigravida woman eat during the day**

A- Only three meals ( )

B- Three main meals in addition to snacks such as fruits and nuts

C- Only two meals ( )

D- I don't know ( )

**10-What is the frequency of drinking adequate water recommended for a primigravida woman to drink daily?**

A- From 2-3 cups ( ) B- From 4-6 cups ( )

C- From 8-10 cups ( ) D- I don't know ( )

**11-Which of the following foods and drinks should be avoided during pregnancy?**

A- Spicy foods ( )

B- Raw or undercooked foods ( )

C- Soft drinks ( )

D- Drinks that contain a high percentage of caffeine such as tea, coffee and cola ( )

E- I don't know ( )

**12-What is the importance of measuring weight regurarly during pregnancy?**

A- To measure the body mass index easily ( )

B- To assess the risks facing the pregnant woman during pregnancy due to weight gain such as high blood pressure and gestational diabetes ( )

C- Underweight may be dangerous to the health of the fetus and affect its healthy growth ( )

D- I don't know ( )

Knowledge of primigravida women about physical activity:

**? 13-What is the importance of phsical activity during pregnancy**

A- Strengthening the muscles of the uterus and abdomen ( )

B- Improving heart health and blood circulation ( )

C- Facilitating the birth process ( ) D- I don't know ( )

**14-Which of the following activities is considered safe for pregnant women?**

A- Kegel exercises ( ) B- Tennis ( )

C- Swimming ( ) D- Walking ( )

E- I don't know ( )

**15-When should you stop exercising?**

A- In case of difficulty breathing and dizziness ( )

B- Slight abdominal pain ( )

C- Feeling hungry during exercise ( )

D- Muscle tension ( )

E- I don't know ( )

**16-How can a pregnant woman continue to exercise safely?**

A- Avoid strenuous and dangerous exercises ( )

B- Follow the instructions of a specialized sports trainer ( )

C- Both A& B ( )

D- I don't know ( )

Knowledge of primigravida women about rest and sleep:

**17-What is the importance of rest and sleep during pregnancy?**

A- Reducing fatigue and stress for the mother ( )

B- Good blood flow to the fetus ( )

C- Escaping problems ( ) D- I don't know ( )

**18-What is the average number of sleep hours suitable for a pregnant woman?**

A- From 5-7 hours ( ) B- From 8-9 hours ( )

C- More than 9 hours ( ) D- I don't know ( )

Knowledge of primigravida women about vaccinations during pregnancy:

**? 19-What are the important vaccinations during pregnancy**

A- Influenza vaccine ( ) B- Rubella vaccine ( )

C- Diphtheria, pertussis and tetanus vaccine ( )

D- Hepatitis B vaccine ( )

E- I don't know ( )

**20-When should a primigravida woman be vaccinated against diphtheria, pertussis and tetanus?**

A- In the first three months of pregnancy ( )

B- In the middle three months of pregnancy ( )

C- In the last three months of pregnancy ( )

D- I don't know ( )

**21-When should a pregnant woman be vaccinated against influenza?**

A- In the third month of pregnancy ( )

B- In the fifth month of pregnancy ( )

C- At any stage of pregnancy ( ) D- I don't know ( )

**22-When should a primigravida woman be vaccinated against hepatitis B?**

A- The first injection six months before pregnancy, and the second injection one month after the first injection Then the third injection six months after ( ),

B- In the eighth month of pregnancy ( )

C- At what stage of pregnancy ( ) D- I don't know ( )

**? 23-What is the importance of vaccinations during pregnancy**

A- Preventing infectious diseases such as measles, mumps and influenza ( )

B- Preventing the transmission of infection to the fetus ( )

C- Both A& B ( )

D- I don't know ( )

Knowledge of primigravida women about pregnancy follow-up:

**? 24-How many visits to the doctor during pregnancy**

A- Once a month during the first three months ( )

B- Once every two weeks during the middle three months ( )

C- Once a week during the last three months ( )

D- Once every two months throughout pregnancy ( )

E- Once a week throughout pregnancy ( )

F- I don't know ( )

**? 25-What is the importance of regular pregnancy follow-up**

A- Early detection of any health problems during pregnancy( )

B- Early detection of genetic diseases ( )

C- To ensure the mother's health only ( )

D- To ensure the fetus's health only ( )

E- I don't know ( )

**26-What are the tests that should be followed up continuously during pregnancy?**

A- Blood pressure test ( ) B- Blood sugar test ( )

C- Urine test ( ) D- I don't know ( )

**27-What are the tests that should be avoided during the first three months of pregnancy?**

( ) A- MRI of the uterus and fetus ( ) B- X-ray

C- CT scan ( ) D- I don't know ( )

Knowledge of primigravida women about safe sexual intercourse during pregnancy:

**28-What is the importance of safe sexual intercourse during pregnancy?**

A- Protecting the spouses from diseases ( )

B- Preventing the transmission of any possible infection to the fetus( ) C- Facilitating the birth process ( ) D- I don't know ( )

? **29-When should sexual intercourse be avoided during pregnancy**

A- In case of vaginal bleeding ( ) B- In case of contractions ( )

C- Both A &B ( ) D- I don't know ( )

**30-What is the importance of early detection of sexually transmitted diseases and vaginal infections?**

A- Protecting spouses from contracting sexually transmitted diseases ( )

B- Obtaining appropriate treatment ( )

C- Protecting the fetus from contracting diseases ( )

D- I don't know ( )

**? 31-What is the source of your information about pregnancy**

A- Personal experience ( ) B- Family ( )

C- Physician ( ) D- Nurse ( )

E- Friends ( ) F- Social media ( )

**Second: Evaluation of the well-being of primigravida women**

**Put a check mark (√) in front of the statement that suits you**

**A-Positive feelings**

| **Never** | **Rarely** | **Sometimes** | **Often** | **Always** | **Items** |  |
| --- | --- | --- | --- | --- | --- | --- |
|  |  |  |  |  | I feel satisfied with my husband's support during pregnancy | 1 |
|  |  |  |  |  | I feel supported by my family for taking care of me during pregnancy. | 2 |
|  |  |  |  |  | I feel satisfied with my experience of health care during pregnancy. | 3 |
|  |  |  |  |  | I feel connected to my child | 4 |
|  |  |  |  |  | I feel very positive about being pregnant | 5 |
|  |  |  |  |  | I feel supported by health care professionals. | 6 |
|  |  |  |  |  | I am happy with my appearance during pregnancy | 7 |
|  |  |  |  |  | Pregnancy has given me a purpose in life | 8 |
|  |  |  |  |  | I care about my health during pregnancy | 9 |
|  |  |  |  |  | I care about my baby's health during pregnancy. | 10 |
|  |  |  |  |  | Pregnancy makes me feel confident. | 11 |
|  |  |  |  |  | I feel happy and psychologically comfortable when I listen to calm music | 12 |
|  |  |  |  |  | I feel pleasure when reading books and magazines to gain information about pregnancy and childbirth | 13 |
|  |  |  |  |  | I feel pleasure when drawing and coloring to express my feelings visually | 14 |
|  |  |  |  |  | I feel satisfied and accomplished when I do handicrafts | 15 |
|  |  |  |  |  | I feel energetic and psychologically comfortable when I go on trips with my family or friends | 16 |

**B-Negative feelings**

| **Never** | **Rarely** | **Sometimes** | **Often** | **Always** | **Items** |  |
| --- | --- | --- | --- | --- | --- | --- |
|  |  |  |  |  | I feel anxious about changing my relationships during pregnancy. | 17 |
|  |  |  |  |  | I feel upset by the physical changes of pregnancy | 18 |
|  |  |  |  |  | I feel anxious about childbirth | 19 |
